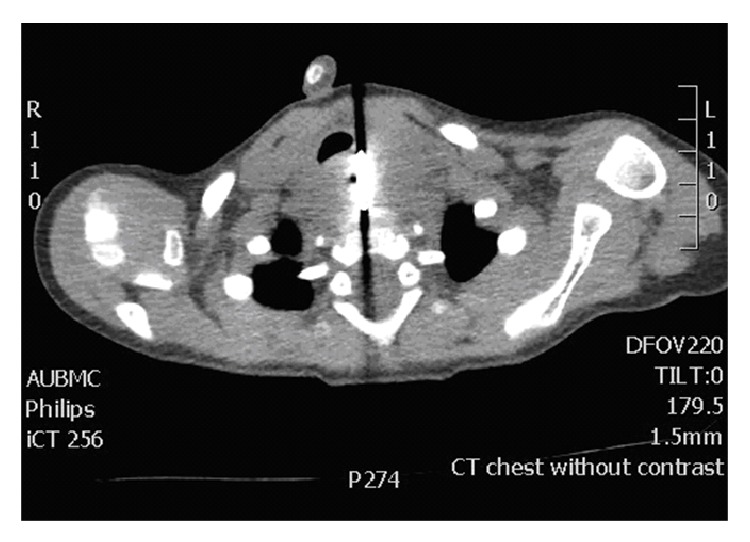# A Case of Battery Ingestion in a Pediatric Patient: What Is Its Importance?

**DOI:** 10.1155/2015/345050

**Published:** 2015-01-27

**Authors:** Elie Alam, Marc Mourad, Samir Akel, Usamah Hadi

**Affiliations:** ^1^Department of Otolaryngology Head and Neck Surgery, American University of Beirut, P.O. Box 11-0236, Riad El Solh, Beirut 1107-2020, Lebanon; ^2^Division of General Surgery, American University of Beirut, P.O. Box 11-0236, Riad El Solh, Beirut 1107-2020, Lebanon

## Abstract

This is a case of a two-year-old boy who has been suffering from food regurgitation and frequent vomiting over the past seven months which were progressively worsening with time. He was initially diagnosed with gastroesophageal reflux disease and treated accordingly but responded only minimally. Investigations and interventional procedures including a chest X-ray showed a metallic round object in the upper esophagus consistent with a button battery which was removed via a thoracotomy after an esophagoscopy was not successful. This child would not have developed such serious complications and would not have required major surgery had the foreign body been identified and removed early on.

## 1. Introduction

Pediatric foreign body ingestion is a problem encountered by many physicians including pediatricians, otolaryngologists, and emergency physicians frequently. Approximately 80% of cases of foreign body ingestions occur in children between the ages of six months and three years [1–3]. Button battery ingestion occurs at an estimate rate of ten in one million people per year, a small group of which are retained in the esophagus and later become complicated [1]. The aim of this report is to describe our case of a pediatric patient who ingested a button battery and was diagnosed late and to highlight the importance of having a high index of suspicion.

## 2. Case Report

This is the case of a two-year-old boy who was referred to our Emergency Department by his pediatric cardiologist for evaluation of his lung condition. The physician was performing a routine echocardiogram for the assessment of the child's preexistent foramen ovale, when he saw a round opacity in the thorax, suspicious of a foreign body. This finding necessitated further evaluation by a chest radiograph.

The patient was hemodynamically stable upon arrival and not in distress. He had normal oxygen saturation and a normal head and neck examination. Examination of the lungs revealed mild crackles over lung bases but with no evidence of stridor or hoarseness.

Upon questioning, the mother reported that he had been having vague upper respiratory symptoms with food regurgitation and frequent vomiting over the past seven months. She denied solid food dysphagia but reported mild daily drooling. These symptoms were progressively worsening over the past four months. He was initially diagnosed with gastroesophageal reflux disease and treated with prokinetics and proton pump inhibitors, to which he responded only minimally.

**Figure 1:**
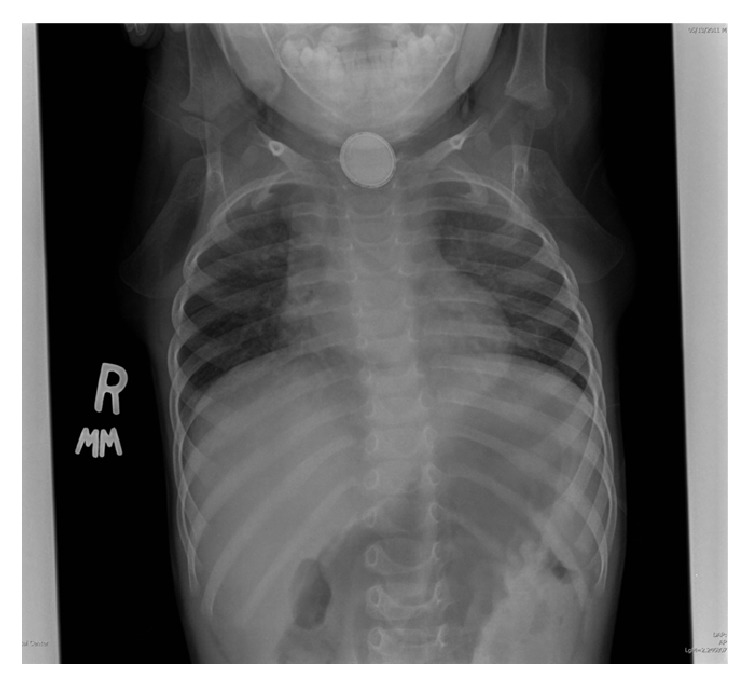


A chest radiograph was done in the emergency room showing the presence of a round metallic density over the topography of the upper esophagus showing irregular contour, with mild mass effect on the left aspect of the trachea (Figure 1). The lung fields appeared clear. Further evaluation by a CT scan showed the same round metallic object at the level of the upper esophagus (Figure 2). A barium swallow was performed and showed that the patient was swallowing without difficulty, with the foreign body apparently separate from the esophageal tract.

The decision was made to perform an esophagoscopy in the operating room to the attempt of foreign body removal. Intraoperatively, the foreign body was not seen but a hard mass was felt at the lateral esophageal wall, which was covered by granulation tissue. Multiple attempts to remove the foreign body were performed but unsuccessful. The decision was made to abort the surgery and proceed with an external approach and the patient was transferred to the pediatric intensive care unit.

Two days later, the patient was scheduled for a right posterolateral thoracotomy and an extrapleural approach for removal of foreign body with esophagostomy and esophagoplasty. The surgery was successful and was followed by a smooth uncomplicated course. The foreign body retrieved was a button battery.

Foreign body ingestion is a frequently occurring problem in pediatric age groups with 75% occurring at ages less than 4 years [4]. Esophageal foreign body impaction (EFBI) is a rare presenting pediatric complaint due to the fact that not all are present immediately following ingestion. The majority of ingested foreign bodies pass through the GI tract with no sequelae; however, those that do cause impaction do so in the upper esophagus, the most common site accounting for more than 75% of all cases [5, 6].

The presenting symptoms can range from being completely asymptomatic to being fatal. In between these ends of the spectrum, symptoms can include GI complaints including vomiting, drooling, dysphagia, odynophagia, and respiratory complaints such as cough, stridor, and choking [1, 7, 8]. However, neither the symptoms upon presentation nor the location of impaction within the esophagus is predictive of the presence of esophageal injury [9].

The complications resulting from ingestion are mainly related to the duration of impaction. Moreover, the type of ingested foreign body affects the complication rate [1, 10]. Many studies have displayed findings that support this conclusion. Denney et al. showed that foreign bodies in situ for more than 24 hours were more likely to cause esophageal ulceration (46%) as compared to those in situ for less than 24 hours (23%) [9]. Similarly Miller et al. concluded that a higher rate of esophageal injury is seen in foreign body ingestion of over one week [11]. There is a wide range of complications that have been reported in the literature. These include mucosal abrasions and lacerations, perforations with mediastinitis, strictures, pulmonary edema, and esophageal diverticulum [1, 10, 12–16].

The child described here ingested a button battery. Previously injury was believed to occur secondary to leakage of alkaline material; however, recent studies proposed that the cause is the passage of a current through the tissue causing hydrolysis of tissue fluids. Moreover, lithium cells have been associated with worse outcomes. This is due to lithium being 3 V cells instead of 1.5 V cells and since they generate more current, more hydroxide is produced and is more rapid than other cells. In addition, studies have shown that the current generates hydroxide at the negative battery pole and as a result the esophageal injury can be predicted by the anatomic location and orientation of the battery [2, 3].

This case highlights the necessity of having high clinical suspicion and intervention early on. Studies have demonstrated that the worst anatomic area of impaction is in the esophagus. Furthermore, there is chance to have injury free removal of an esophageal battery if removed within 2 hours of ingestion [3]. The child described above would not have developed such serious complications and would not have required major surgery had the foreign body been identified and removed early on. As a result, physicians who are caring for children who present with respiratory or GI complaints should keep a high index of suspicion of foreign body ingestion especially if the child is nonverbal. In addition, new emerging technologies discuss battery coating which if swallowed decreases the external electrolytic currents which cause tissue injury. The authors conducted animal studies and reported significant decrease in tissue injury compared with uncoated control batteries [17]. More importantly, parents of young children should take extra caution in storing items which could be ingested by children around the house. Small items especially ones that have chemical composition such as batteries should be kept in areas out of reach of children to insure they never have access to them.

## Figures and Tables

**Figure 2:**